# Mycobacteria-Based Vaccines as Immunotherapy for Non-urological Cancers

**DOI:** 10.3390/cancers12071802

**Published:** 2020-07-05

**Authors:** Estela Noguera-Ortega, Sandra Guallar-Garrido, Esther Julián

**Affiliations:** 1 Mycobacteria Research Laboratory, Department of Genetics and Microbiology, Faculty of Biosciences, Universitat Autònoma de Barcelona, Bellaterra, 08193 Barcelona, Spain; sandra.guallar@uab.cat; 2 Center for Cellular Immunotherapies and, Division of Pulmonary, Allergy, and Critical Care, Department of Medicine, Perelman School of Medicine, University of Pennsylvania, Philadelphia, PA 19104, USA

**Keywords:** BCG, non-tuberculous mycobacteria, adjuvant, immunotherapy, mycobacteria antigens

## Abstract

The arsenal against different types of cancers has increased impressively in the last decade. The detailed knowledge of the tumor microenvironment enables it to be manipulated in order to help the immune system fight against tumor cells by using specific checkpoint inhibitors, cell-based treatments, targeted antibodies, and immune stimulants. In fact, it is widely known that the first immunotherapeutic tools as immune stimulants for cancer treatment were bacteria and still are; specifically, the use of *Mycobacterium bovis* bacillus Calmette-Guérin (BCG) continues to be the treatment of choice for preventing cancer recurrence and progression in non-invasive bladder cancer. BCG and also other mycobacteria or their components are currently under study for the immunotherapeutic treatment of different malignancies. This review focuses on the preclinical and clinical assays using mycobacteria to treat non-urological cancers, providing a wide knowledge of the beneficial applications of these microorganisms to manipulate the tumor microenvironment aiming at tumor clearance.

## 1. Introduction

The use of immunotherapy for cancer treatment was reported around 130 years ago, with the use of the inactivated product from two strains of bacteria, *Streptococcus pyogenes* and *Serratia marcenses*, namely Coley’s toxin, which were mixed in order to be injected into patients affected by diverse types of cancer. In a series of experiments, Dr. Coley demonstrated the effect of the product in the reversion of cancer in treated patients [1,2,3]. After these first successful works, mycobacteria entered into the immunotherapy scene owing to the confluence of different facts. On the one hand, at that time, Dr. Pearl observed an inverse relationship between deaths due to tuberculosis (TB) and those due to cancer in a series of autopsies. On the other hand, during the second decade of the last century, the perseverance of doctors Albert Calmette and Camille Guérin led to the development of an attenuated strain in animal models derived from an original isolate of *Mycobacterium bovis* that was later called *Mycobacterium bovis* bacillus Calmette-Guérin (BCG). As soon as BCG was considered a safe vaccine, and along with the successful experience using Coley’s toxin, and in view of the evidence from Pearl’s research, several trials were conducted in the fight against different types of cancer using BCG. Since the 1930s, studies in gastric cancer patients [4], melanoma [5], and leukemia [6] treated with BCG showed disease remission or a non-relapsing disease. Despite the appearance and the rapid and broad implementation of chemotherapy and radiotherapy for cancer treatment, the study of mycobacteria as an immunotherapeutic agent was not abandoned [7]. Thus, in the 1970s and 1980s, BCG was used by injecting into the tumor or in combination with other therapies in melanoma [8,9], as well as in lung [10,11,12], cervical [10,13], ovarian [14,15], colon [16], and head and neck cancers [10], etc.

All these years of preclinical and clinical assays finally resulted in the use of BCG as immunotherapeutic agent for the treatment of non-muscle invasive bladder cancer (NMIBC). This type of tumor only affects the mucosa or sub-mucosa of the bladder wall. When mycobacteria are intravesically instilled, the bladder cavity provides the optimal conditions for an efficacious BCG effect due to it being a closed space. Although BCG is an attenuated strain and belongs to biosafety level 2 due to its ability to cause infections in some cases, it can be instilled in its live form into the bladder because, since it is a closed space, BCG could arduously spread to the patient body. Furthermore, and as the bladder cavity enables mycobacteria to be in close contact with the tumor, this interaction seems to be necessary for a favorable outcome. After some successful trials conducted by Dr. Morales in 1976, BCG was later approved for the treatment of NMIBC by the Food and Drug Administration (FDA), and until this day is the first option of treatment for these patients. It is worth mentioning that BCG is the most efficacious treatment to avoid recurrences and progression of NMIBC, even superior to intravesically instilled chemotherapeutic drugs.

## 2. Use of other Species Different from BCG

Although BCG has been the most studied mycobacteria for cancer treatment, other species or antigens derived from mycobacteria species have been studied for the treatment of urological and non-urological malignancies showing also promising immunotherapeutic properties. Those are, for instance, *Mycolicibacterium smegmatis* (basonym *Mycobacterium smegmatis*), *Mycolicibacterium phlei* (basonym *Mycobacterium phlei*), *Mycolicibacterium vaccae* (basonym *Mycobacterium vaccae*), *Mycolicibacterium obuense* (basonym *Mycobacterium obuense*), “*Mycobacterium indicus pranii*”, *Mycolicibacterium brumae* (basonym *Mycobacterium brumae*), or more recently *Mycobacterium paragordonae*, and an attenuated strain of *Mycobacterium tuberculosis*.

In depth, *M. smegmatis* was first isolated by Lustgarten in 1885 from genital secretions (smegma) in a patient with a penile ulcer [17]. *M. phlei*, found in the environment [18], was first described in 1899, called the grass bacillus of Timothy bacillus. Since their discovery, *M. smegmatis* and *M. phlei* have been used as models for the study of mycobacteria, and in consequence, *M. phlei* was one of the first species considered for cancer treatment. Some cases of infections due to *M. phlei* are described in the literature [19,20,21,22,23,24,25,26], but studies using *M. phlei* for cancer treatment are based on non-viable mycobacteria. The formulation based on *M. phlei*, known as *M. phlei* cell wall-nucleic acid complex (MCNA), or commercially known as Urodicin^TM^, consists in a preparation of cell wall (CW) fractions plus DNA of the same bacterium [27]. 

Four more mycobacteria are used in non-viable form for cancer treatment. The *M. vaccae* strain used for cancer treatment is the R877R (NCTC 11659), which is a selected rough colony variant of a strain originally isolated from the Ugandan environment [28]. The commercial preparation is called IMM-201, previously SRL172. *M. obuense* was initially isolated from soil samples and the sputum of a patient with lung disease in 1971 [29]. *M. obuense* is closely related to *M. vaccae* [30], and the specific strain used for cancer treatment (NCTC 13365 strain) is commercially called IMM-101. *Mycobacterium indicus pranii*, called MIP, (DSMZ 45239T) is a saprophytic bacterium that has been extensively used as an immunotherapeutic agent for leprosy treatment but is currently under evaluation for the treatment of other infectious diseases and other kind of illnesses [31]. Finally, *M. paragordonae* (Mpg) is a slow-growing mycobacterium that fails to grow above 37 °C and is isolated from water supply systems worldwide [32,33]. This mycobacterium has recently been proposed as a novel live vaccine candidate for the prevention of mycobacterial infections, showing enhanced protective efficacy in vaccinated mice compared to BCG [34].

Finally, two mycobacteria have been studied in pre-clinical studies in their live form. *M. brumae* was isolated from soil and water samples in 1993 by M. Luquin et al. [35], and similarly to the above-mentioned species, is a rapid grower. No cases of infections due to *M. brumae* have been described, and its non-pathogenicity and non-toxicity have recently been demonstrated [36,37]. *M. brumae* has shown to inhibit tumor growth and increase survival in preclinical studies for bladder cancer (BC) treatment [38,39,40,41,42]. Finally, MTBVAC, a Spanish vaccine developed by deleting virulence factors of *M. tuberculosis* and currently in clinical trials for TB prevention [43], has been analyzed for its value in BC treatment [44].

**Figure 1:**
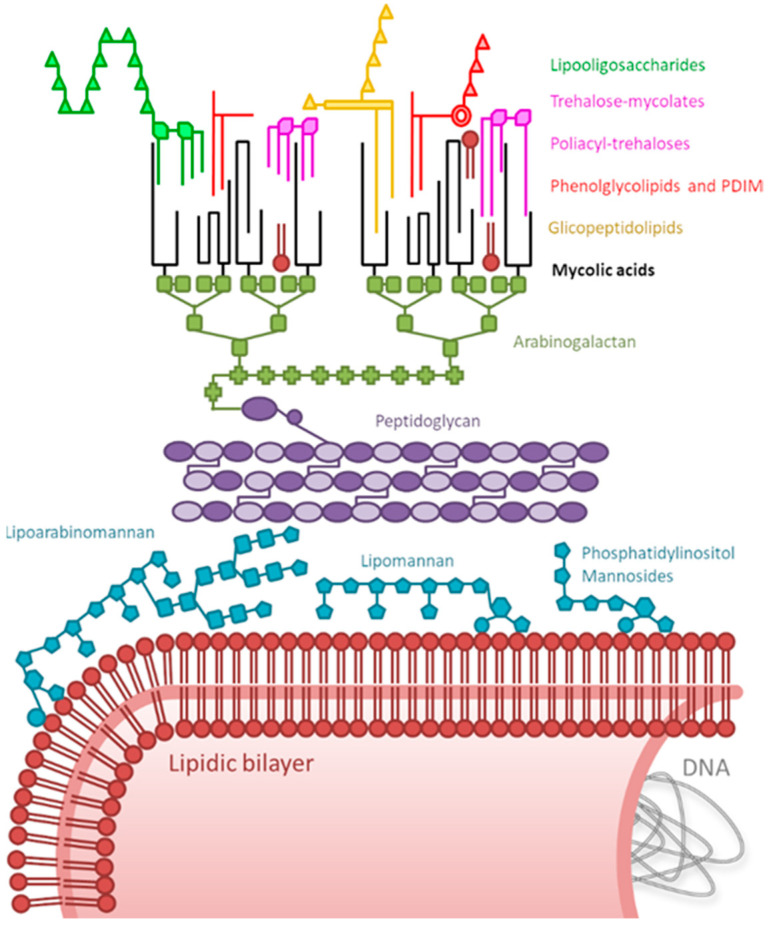
Schematic representation of mycobacterium immunomodulatory molecules. The peptidoglycan–arabinogalactan–mycolic acids complex is covalently linked to the cell membrane and is present in all mycobacteria. Glycopeptidolipids, lipooligosaccharides, phenolglycolipids, phthiocerol dimycocerosates (PDIM), and poliacyl-trehaloses are located in the outer membrane of only some species. Mycolic acids, lipomannan, and trehalose-mycolates are present in all mycobacteria, but the fine structure differs among different species. Neither the cell nor the whole cell wall molecules are drawn to scale.

Not only the whole mycobacteria, but also some cell extracts or purified mycobacteria antigens from these species, have been studied in preclinical and clinical studies for cancer treatment. Despite the fact that the molecule or molecules of BCG responsible for its antitumor effect are still unknown, several genus-specific antigens have been described in mycobacteria cells, and most of them are known stimulators of the immune system [45,46]. Antigens belonging to different cell fractions are recognized by surface-located receptors present both in professional and non-professional antigen-presenting cells. Most of the structural molecules located in the mycobacterial cell wall (Figure 1) are agonists of immune receptors such as lectins (Mincle, Dectin 1, or mannose receptor), Toll-like receptors (TLR1, TLR2, TLR6), or T cell receptors via CD1 (detailed in reference [46]). Signaling through these receptors can induce the production of cytokines and/or chemokines favoring a desirable pro-inflammatory profile in the tumor micro-environment [47,48,49].

Due to the success of BCG in the treatment of NMIBC, most of the research in the last thirty years for the use of mycobacteria in cancer treatment has been carried out on urological cancers (reviewed in [41,46,50]). However, in view of the comprehensive research already done, and the interest in these agents for combining with other antitumor therapies, we will focus our review on the use of mycobacteria or mycobacteria components for the treatment of non-urological cancers (see Table 1). In all these cancers, there is still not a case in which mycobacteria or their components became the standard of care, as in the use of BCG in NMIBC.

## 3. Melanoma and Mycobacteria

**Figure 2:**
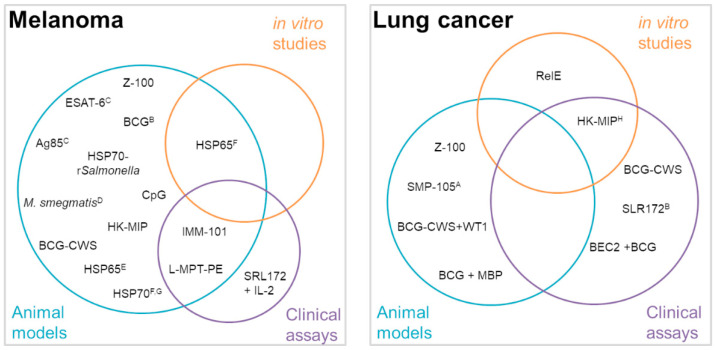
In vitro and in vivo studies using mycobacteria-derived agents carried out for the treatment of melanoma and lung cancer. ^A^, alone or in combination with chemotherapy; ^B^, along with MUC1-MBP fusion protein; ^C^, B16 cells expressing mycobacterial antigens; ^D^, in combination with monosodium urate crystals; ^E^, anchored to tumor proteins; ^F^, administered together with B16 cell lysates; ^G^, co-expressed with herpes simplex thymidine kinase in *Salmonella typhimurium*; ^H^, in combination with chemotherapy and radiotherapy. Ag85—antigen 85 complex, BCG—*Mycobacterium bovis* bacillus Calmette-Guerin, BEC2—anti-idiotypic antibody mimicking the GD3 ganglioside expressed on most small-cell lung cancer (SCLC) cells, CpG—cytosine-guanosine nucleotide, CWS—cell wall skeleton, HK—heat-killed, HSP—heat-shock proteins, IL-—Interleucin, L-MTP-PE—liposomal muramyl tripeptide phosphatidyl ethanolamine, MBP—maltose binding protein, MIP—*Mycobacterium indicus pranii*, RelE—toxin of *Mycobaterium tuberculosis*, SMP-105—CWS-BCG Tokyo 172, SRL 172—*M. vaccae* commercial preparation, WT1—Wilms’ tumor protein, Z-100—an arabinomannan from *M. tuberculosis* strain Aoyama B.

After NMIBC, melanoma is the cancer in which more mycobacterial products have been studied as antitumor agents. Around three dozens of these studies described purified antigens, cell fractions, or whole mycobacteria as possible therapies for melanoma (summarized in Figure 2).

### 3.1. Purified Antigens and Cell Extracts

Many studies focused on the use of the immunogenic mycobacterial heat-shock proteins (HSP). Studies on *M. tuberculosis* HSP70 have shown contradictory results. On the one hand, HSP70 protein administered together with B16 melanoma cells lysate to tumor-bearing mice suppressed tumor growth and metastasis, and extended mice survival [51]. Furthermore, an attenuated *Salmonella typhimurium* that co-expresses HSP70 and herpes simplex virus thymidine kinase genes showed inhibitory effects on melanoma growth and prolonged tumor-bearing mice survival [52]. On the one hand, HSP70 was found to be immunosuppressive in an allograft model by reducing rejection of the implants [53]. Moreover, the Prostate-Specific Antigen (PSA)–HSP70 fusion protein was as immunostimulating as PSA alone in a murine melanoma model [54].

Another HSP tested for melanoma was BCG HSP65. Cytosine-guanosine nucleotide (CpG) enhanced the antitumor effect of BCG HSP65 when anchored to tumor antigens Her2 [55] or tumor-associated antigen Mucin 1 (MUC1) [56,57], both expressed in melanoma cells. In fact, mycobacterial DNA alone was also shown to have therapeutic properties against melanoma. CpG oligodeoxynucleotides from *M. bovis* encapsulated in liposomes showed antitumor activity against B16 tumor-bearing mice when administered into the tumor [58]. Another studied combination is BCG HSP65 and melanoma cell lysates. When administered together, they induced apoptosis in different melanoma cell lines and inhibited tumor growth in tumor-bearing mice [59]. As evidence of the concept for an immunotherapy consisting of inducing the transient expression of mycobacterial HSP65 or the antigen 85 complex (Ag85) (fibronectin-binding protein) to make melanoma tumors more immunogenic, Tarrant et al., induced subcutaneous tumors using B16-F10 cells transfected with either HSP65 or Ag85 and observed a significant tumor burden reduction in the case of Ag85-B16-F10 tumors but not in the tumors that expressed HSP65, indicating that Ag85 protein is more immunogenic [60]. A completely different strategy is the use of tumor cells transfected to express Ag85 as a vaccine. The addition mycobacterial agent enhances the immune response against the parental cell line and protects mice form developing tumors [61].

The ESAT-6 antigen of *M. tuberculosis* has also been used in melanoma studies as prophylactic or therapeutic treatment. The prophylactic strategy consisted of transfecting B16 cells to express ESAT-6 fused to glycosyl-phosphatidylinositol (GPI) and IL-21. In these experiments, vaccinated mice showed significantly reduced tumor and lung metastasis [62]. This antitumor response was even more enhanced when animals were previously primed with an ESAT-6-GPI DNA vaccine [63]. Therapeutic efficacy was evaluated by administering ESAT-6-anchored IL-2 or IL-12 DNA vaccines into the tumor, with the first combination being able to completely remove tumors [64]. The same authors were successful in a second therapeutic strategy. When they used exosomes from B16-ESAT-6 transfected cells that contained antigens from both the tumor and the mycobacterium to treat melanoma-bearing mice, they observed a significant reduction in tumor growth [65]. The mechanism of action of these exosomes is immediate due to the activation of macrophages and dendritic cells (DCs) followed by the suppressive effects of the adaptive immunity responding against ESAT-6 [66].

Different CW structural antigens have been evaluated. Z-100, an arabinomannan from *M. tuberculosis* strain Aoyama B, known to establish a balance of Th1/Th2 responses in tumor-bearing mice [67], has been tested as immunotherapy in highly metastatic melanoma mouse models. Monotherapy or combined therapy with radiation inhibits lung and lymphatic metastasis [68,69,70]. A synthetic peptidoglycan mimicking muramyl dipeptide from BCG with an additional peptide and encapsulated in liposomes: liposomal muramyl tripeptide phosphatidyl ethanolamine (L-MTP-PE) [71] has been widely studied in different types of cancers. L-MTP-PE showed antitumor activity in dogs suffering spontaneous early-stage oral melanoma, but was not efficacious in advanced-stage melanoma [72], as had been already observed in a clinical trial in stages III and IV melanoma patients [73]. A fusion protein consisting of the maltose-binding protein (which binds to Toll-like receptor-2 (TLR2) and MUC1 demonstrated higher antitumor activity in B16 MUC1-expressing cells when BCG was present [74].

Regarding mycobacterial cell fractions, squalane emulsified cell wall skeleton (CWS) from BCG Tokyo (BCG-CWS) has been mainly used as adjuvant for melanoma vaccines. BCG-CWS significantly lowered lung metastasis events in B16-BL6 melanoma subline cells bearing mice administered both intravenously and subcutaneously, due to its effect on reducing tumor growth and inhibiting angiogenesis [75]. On the contrary, when MIP-CWS was tested in B16-F10 melanoma-bearing mice and due to its capacity to induce Th2, a worse antitumor effect was obtained compared to MIP-CW [76] or whole MIP [77] (see Section 3.2.).

### 3.2. Whole Mycobacteria

The whole cells of *M. vaccae*, *M. obuense*, MIP, *M. smegmatis*, and BCG have been studied for melanoma. SRL172 showed a positive correlation between longer survival and intracellular IL-2 detection in peripheral blood lymphocytes when used as antitumor agent for the treatment of stage IV melanoma patients [78]. Then, a clinical trial was performed administering an SRL172 plus IL-2 combination therapy, but only three partial remissions were observed in a total of 16 patients. Thus, no therapeutic effect was established [79]. Nevertheless, a retrospective study concluded that this treatment could be effective in lower grade melanoma patients [80].

*M. obuense* was unsuccessfully used in two clinical trials. Although IMM-101 had a positive effect on avoiding metastasis in a melanoma mouse model, it showed no effect on the overall tumor burden (NCT01559818, NCT01559819) [81]. Furthermore, in a small clinical trial with advanced melanoma, patients tolerated the therapy, but only 15% of clinical responses were observed (NCT01308762) [82]. Recently, immune checkpoint inhibitors have become the standard of care for melanoma patients, as they show the highest long-term survival of patients with metastatic melanoma that has ever seen with any other therapy, although high levels of toxicity have been observed. Dalgleish and collaborators, in a small clinical trial, showed it was more efficacious without added toxicity when combining IMM-101 and checkpoint inhibitors [83].

MIP preparation has been also assayed in subcutaneously implanted B16-F10 melanoma-bearing mice and reduced tumor growth by triggering macrophage and DCs activation and increasing T-cell infiltration inside the tumor [77]. MIP activates Th1 immune response through TLR2 and MyD88 axis [84] and inhibits invasion of the highly invasive B16-F10 cells [85].

Not only can proteins, lipids, and sugars be immunogenic: insoluble salts like urate also are. Accumulation of crystallized uric acid in the joints triggers an inflammatory response, resulting in an attack of gout. A combination therapy with monosodium urate crystals and *M. smegmatis* showed a delaying effect in melanoma tumor growth, unlike each monotherapy. In this case, the antitumor efficacy correlated with the presence of monocyte-derived DCs in the tumor-draining lymph nodes [86].

Antitumor effect of intra-lesional BCG Tice was also tested. BCG triggered antitumor effect by changing the melanoma microenvironment due to a switch from M2 to M1 macrophages [87]. Moreover, this treatment also attracted important antitumor immune cells, such as ɣδ T cells [88]. Despite these promising results, unfortunately, not even combining BCG with checkpoint inhibitors [89] resulted in clinical benefit in a phase I study which involved advanced metastatic melanoma patients. Dose escalation was not well tolerated and triggered immune-related adverse events [90]. Thus, after all the attempts to find an efficacious mycobacterial immunotherapy for melanoma patients, there is still no consensus treatment yet [91].

## 4. Lung Cancer and Mycobacteria

In the case of lung cancer (LC), purified mycobacteria antigens, BCG extracts, MIP, and *M. vaccae* have been studied as immunotherapeutic agents.

### 4.1. Purified Antigens and Cell Extracts

Mycobacteria and purified antigens have been tested in the Lewis lung carcinoma (LLC) mouse model. The Z-100 antigen in combination with radiotherapy significantly reduces both primary tumor growth and pulmonary metastasis of LLC-bearing mice, being more effective than each therapy individually [92]. Not only polysaccharides but also the *M. tuberculosis* toxin RelE showed an inhibitory effect on human A549 lung adenocarcinoma tumor cells [93].

BCG extracts have also been used combined with other treatments for LC therapy. The SMP-105 formulation, consisting of CWS purified from BCG Tokyo 172 strain, in combination with mitomycin C-inactivated LLC cells, had a therapeutic effect and induced an IFN-γ response through the activation of TLR2, and independently from TLR4, in LLC-bearing mice [94]. Moreover, the intradermal administration of BCG-CWS, combined with Wilms’ tumor protein (WT1), in mice, induced a cytotoxic immune response against LC cells expressing WT1 [95]. BCG-CWS-treated non-small-cell LC (NSCLC) patients showed prolonged survival rates without worsening their quality of life. However, in the case of small-cell LC (SCLC) patients, although a small clinical trial suggested longer survival and relapse-free survival in patients treated with an immunoadjuvant consisting of BCG extract bound to hydroxyapatite and microparticulated tuberculin called CalTUMP in combination with BEC2 (which is an anti-idiotypic antibody mimicking the GD3 ganglioside expressed on most SCLC cells) [96], two other studies demonstrated no beneficial effect of the combined therapy [97,98].

### 4.2. Whole Mycobacteria

Most studies in lung used MIP and SRL172, but one study used whole BCG. Although maltose-binding proteins from *Escherichia coli* have no direct inhibitory effect on the LLC cell line, in combination with BCG they have a synergic effect on LLC-bearing mice [99].

A recent study showed the antitumor effect of heat-killed (HK) MIP on several cancer cell lines, including A549 cells. However, neither live MIP nor supernatant from MIP cultures showed an inhibitory effect on cancer cells [100]. However, MIP showed a beneficial effect in the past as an adjuvant in the therapy of NSCLC patients. When MIP was administered along with cisplatin chemotherapy and radiotherapy therapy, patients were able to finish the treatment and experienced a regression in tumor size and improvement in the lung function compared to the control group, which did not receive MIP [101]. Furthermore, intradermally administered MIP along with paclitaxel and cisplatin treatment increased the overall survival and the progression-free survival compared to the chemotherapy alone group [102].

In view of the promising results of SRL172 in prostate cancer [103,104] and melanoma patients (see Section 3.2.), two phase II clinical trials were performed using it in LC patients in which a small group of NSCLC [105] and SCLC patients [106] received chemotherapy together with intradermal injections of SRL172. No differences regarding toxicity were observed between the chemotherapy alone or the combination treatment in any group and, in both cases, there was a trend towards the improvement of the median survival and the quality of life of the patients [107]. Only in the case of NSCLC patients, a trend towards improvement of the response rate was observed on SRL172-treated patients. The phase III trial on a cohort of more than 400 NSCLC patients confirmed that SRL172 led to the improvement in the quality of life of the patients, but without any improvement on overall survival [108]. However, the number of intradermal SRL172 injections was very variable between patients in this study. When the data obtained were re-analyzed taking into account the histological lung tumor type and the number of adjuvant inoculations, adenocarcinoma patients, but not squamous cell carcinoma patients, survived significantly longer when receiving combination therapy [109]. A more recent study on the mechanistic effect demonstrated that SRL172 showed capacity to activate γδ T-cells to be cytotoxic to A549 cell line [110].

## 5. Gastrointestinal Tract Cancers and Mycobacteria

Mycobacteria agents have been tested in several parts of the gastrointestinal tract from the esophagus to the rectus, including the stomach and pancreas.

*M. smegmatis* is the only mycobacterium studied for the treatment of esophageal tumors. *M. smegmatis* was used as a vector to produce a recombinant bacterium containing MAGEA3 and SSX2, two important immunogenic tumor antigens. Treatment of esophageal tumor-bearing mice with the recombinant mycobacterium inhibited tumor growth and reduced the tumor weight and volume [111].

In a study of locally advanced gastric cancer in a group of 156 patients, the administration of BCG Moreau plus chemotherapeutic treatment up to two years after resection of tumors resulted in a significant increase in survival with respect to the monotherapies [112].

Several studies have been carried out in pancreatic cancer using mycobacteria, mainly in combination with chemotherapeutic agents. In fact, it has been demonstrated that gemcitabine (the standard chemotherapeutic agent for this type of cancer) enhanced the antitumor effect of cytotoxic T cells induced by BCG-stimulated DCs, by sensitizing pancreatic tumor cells [113]. Using purified mycobacterial antigens, a vaccine composed of mycobacteria HSP65 plus Panc02 pancreatic cancer tissue lysate was inoculated in mice before implanting Panc02 cancer cells, showing prolonged survival in treated tumor-bearing mice compared to unvaccinated mice [114]. The most successful mycobacteria immunotherapy for this type of cancer was achieved using IMM-101, which was designed as an orphan drug in 2014 for the treatment of advanced pancreatic cancer patients. Repeated treatment with IMM-101 plus chemotherapy (over a year), showed significantly enhanced survival rates compared to patients receiving only chemotherapy [115]. The recent evaluation of the administration of IMM-101 plus gemcitabine in advanced pancreatic cancer patients was shown to be as safe as chemotherapy alone and suggests improved survival rates [116]. Although HK-*M. obuense* showed great success in the treatment of pancreatic cancer patients, little was known about its cytotoxic and immunomodulatory activity. Recently, Bazzi et al. published an extensive work on how IMM-101 modulates DC, macrophages, and granulocytes, in terms of surface markers and cytokine secretion profile [117,118].

Regarding colorectal cancer, different strategies have been designed for the use of mycobacteria components. In vitro experiments have shown that exposure of HCT116 colon cancer cells to BCG Pasteur-CWS before ionizing radiation resulted in increased cell death in a caspase-independent manner, through the induction of autophagic cell death mediated through TLR2 and TLR4 signaling [119]. Another group reported that BCG Tokyo-CWS significantly reduced lung metastasis in a colon26-M3.1 carcinoma mouse model [75]. The adjuvant DETOX (*M. phlei*-CW together with *Salmonella minnesota* lipid A) included in an allogeneic cell vaccine plus subcutaneous IL-1 treatment in metastatic colorectal carcinoma patients was shown to be safe, also suggesting a beneficial effect [120]. The *M. tuberculosis* Rv2299c (which belongs to the HSP90 family) has been used alone or in combination with tumor antigen-activated DC and enhanced reduced tumor burden in a colon cancer mouse model, but only the combination induced a long-term systemic response [121]. Finally, the use of IMM-101 first for priming before tumor challenge, and later as a subcutaneous therapeutic agent in colorectal mouse models, showed a significant reduction in the number of lung metastatic lesions. However, IMM-101 on its own administered subcutaneously as therapy had no significant effect on tumor growth [81]. Some clinical trials demonstrated the efficacy of repeated injections (up to a year) of IMM-101 in colorectal cancer (NCT03009058). In combination with radiation in metastatic colorectal patients in which other therapies were not effective, no synergy was observed (NCT01539824). Finally, the use of the whole HK-*M. paragordonae* alone, or in synergism with cisplatin in tumor-bearing mice, improved mice survival rate and enhanced a strong activation of the immune system through natural killer (NK) and DCs activation, and release of cytokines such as IL-12 or tumor necrosis factor (TNF-α) [122].

Regarding hepatoma treatment, different vaccines have been designed in which some peptides of mycobacteria, HSP70 [123,124], or *M. bovis* HSP65 [125,126] have been included in the composition. After treating hepatoma mice models, a beneficial effect was observed by delaying the progression of liver tumors, which was mediated by the induction of favorable antitumor immunity. Moreover, the 64-kDa protein from BCG, which shares antigenic determinants with line 10 hepatoma, Meth A, CT-26, and RL female 1 mouse tumor cells, showed antitumor activity in immunized guinea pigs [127] and also in immunized mice [128]. Studies have also been carried out on mycobacteria extracts. In vitro studies showed a growth inhibition effect of the optimized formulation of SMP-105 in Line 10 hepatoma cells [129]. SMP-105 treatment close to the tumor focus in guinea pigs bearing syngeneic line 10 hepatoma was able to eliminate an established tumor and metastasis [130]. Repeated intra-lesion injections of DNA from BCG plus poly-L-lysine resulted in a delay of tumor growth and complete cure of guinea pigs inoculated with line 10 hepatoma cells, while both monotherapies were ineffective [131]. Finally, intra-tumor vaccination using CalTUMP combined with proton-beam radiotherapy has shown to be safe and may be a promising treatment for recurrence reduction [132].

## 6. Breast Cancer and Mycobacteria

Different vaccines using recombinant BCG were evaluated for prevention or treatment of breast cancer. Most of those studies are based on BCG expressing MUC1 and adding or co-expressing some other immunostimulatory molecules, such as cytokines or chemokines, in order to improve the relatively poor immunogenicity of this breast-tumor-associated antigen. A human IL-2-MUC1-expressing-BCG (Pasteur strain) vaccine was used to immunize humanized mice that were later challenged with ZR75-1 human breast cancer cells, showing a reduction of palpable tumors together with CD8+ T cells immune response [133]. Immunization with recombinant BCG expressing MUC1 and granulocyte-macrophage colony-stimulating factor (GM-CSF) inhibits breast tumor growth in mice [134]. BCG expressing variable-number tandem repeats of MUC1 and CD80 inhibits tumors in mice, triggering the infiltration of CD4 and CD8 lymphocytes and the production of IFN-γ in splenocytes [135]. Furthermore, BCG plus allogenic tumor cells vaccine has shown to be safe in mice and patients with advanced breast cancer and, combined with chemotherapy and radiotherapy, showed to be practicable [136,137].

Other vaccines showed efficacy in breast cancer. Ag38-expressing cancer cells administered with IL-12 protected against the formation of mammary tumors [138]. Immunotherapeutic treatment with DNA vaccines that contain BCG HSP65 has been also evaluated for breast cancer treatment in mice, showing an effect on reducing tumor growth [139,140,141]. SRL172, BCG, and *M. phlei* CW extracts have also been studied. SRL172 plus anti-HER-2 antibodies stimulate the cytotoxic activity of immune cells to tumor HER2-expressing cells [142]. Intra-tumor injection of BCG (Frappier strain, named PACIS^MD^) or *M. phlei*-CW extract combined with photodynamic therapy increases the antitumor effect of photodynamic therapy alone by inducing the infiltration of immune cells, which favors the antitumor response [143].

The use of MIP has also been proposed in the literature to resolve breast cancer cases. MIP plus Survivin and alum immunization before tumor implantation seemed to induce a long protective effect [144]. More recently, MIP combined with 1’-S-1’-acetoxychavicol acetate and cisplatin reduced tumor volume of 4T1 tumors in mice with no observed toxicity, which is enough evidence to set up a clinical trial [145]. As observed in a melanoma mouse model (Section 3.2.), *M. smegmatis* plus monosodium urate crystals reduced the growth rates in the orthotopic 4T1 tumors and decreased the 4T1 lung metastases. [86].

## 7. Ovarian and Cervix Tumors and Mycobacteria

From the beginning of the 1990s, there were no studies published on the antitumor potential of mycobacteria or mycobacteria components for ovarian or cervix tumors. In 2009, a study including 73 ovarian cancer patients revealed that the efficacy of intracutaneously administered BCG-CWS in different stages of ovarian cancer patients is compromised in women who underwent lymphadenectomy compared to patients who did not [146]. In 2012, Gottschalk and collaborators demonstrated the immunostimulatory effect of the 30kDa antigen (or phosphate transport protein (Pst-1)) from *M. tuberculosis* on the cytotoxic function of NK on five ovarian cancer cell lines through monocyte activation in a cell–cell contact-dependent manner [147]. In 2014, Yuan et al. constructed a fusion protein consisting of HSP70 plus a single-chain antibody variable fragment which makes the protein specifically target mesothelin-expressing tumors such as mesothelioma or ovarian cancer. The construct improved DC antigen presentation, which resulted in inhibited tumor growth and prolonged survival in orthotopic mouse models of papillary ovarian cancer [148].

Using the unique opportunity of the fact that human papillomavirus type 16 (HPV-16) expresses E7 protein in HCP-linked cervical tumors, many immunotherapy strategies have been based on the combination of highly immunogenic mycobacterial proteins and E7 protein. For instance, a suicidal plasmid containing HSP70 and E7 induced significantly higher cytotoxic T cell immune responses against E7-expressing tumors than the plasmid only containing E7 [149]. A similar approach using a fusion protein between HSP70 with E7 gene induced an immune response and a prolonged survival of TC-1 cervical cancer model tumor-bearing mice [150].

Like *M. tuberculosis* HSP70, HSP65 from *M. bovis* has been fused to E7 to treat cervical tumors. Two studies have demonstrated the efficacy of this fusion protein in mouse models, in which tumor regression was observed due to the triggered CD4-mediated immune response [151,152]. Finally, a synthetic chimeric peptide from computationally predicted HPV-16 T cell epitopes was used to immunize E6- and E7-expressing-TC-1-bearing mice along with different adjuvants including HK MIP, demonstrating therapeutic and protective activity. However, the peptide in combination with MIP showed less effect, both in vitro and in vivo, than the other adjuvants used in the study [153]. Two other posterior studies tested its efficacy in the clinics. Both of them concluded that the HSP65-E7 fusion protein (SGN-00101) confers a benefit for cervical intraepithelial neoplasia III patients [154,155].

Whole mycobacteria MIP and BCG cells have also been tested in cervical tumors. The antineoplastic potential of live BCG Tokyo was tested on HeLa cells, with an inhibitory effect being observed [156]. Similarly, whole HK MIP had a cytotoxic effect on CaSki cervical cancer cells, while not on non-cancerous cells [100].

## 8. Hematopoietic and Lymphoid Malignancies and Mycobacteria

There are reports in the literature on the use of specific mycobacterial antigens, cell extracts, and whole mycobacteria cells against leukemia. HSP70 transfected into L1210 mouse leukemia cells showed reduced tumorigenicity in immunocompetent mice, and HSP70-L1210 cells also reduced tumor burden when used either as vaccine or treatment in the L1210-parental mouse model [51,157]. Another approach is vaccinating using BCG-CWS plus WT1 peptide also expressed in leukemia, which demonstrated that mycobacteria components increase WT1-specific immune responses, favoring rejection of WT1-expressing leukemia cells [95]. Finally, intradermal injection of MIP in BALB/c and C57BL/6 mice with Sp2/0 (myeloma) and EL4 (thymoma) cells, respectively, inhibited tumor growth while triggering an antitumor immunity. The contribution of IFN-γ, CD4, and CD8 T cells is critical on MIP effect [158].

Many vaccines comprising mycobacteria HSPs have been studied for lymphoma treatment. Vaccination with HSP70 plus idiotype-encoding plasmids in the 38C13 murine lymphoma model enhanced survival rates in tumor-bearing mice [159]. Vaccination with A20 B cell lymphoma cells plus HSP70 together with anti-idiotype before tumor induction in the A20 murine lymphoma model triggered antitumor immunity by activating tumor cytotoxicity in T cells and increasing IFN-γ secretion and anti-A20 IgG2a levels [160]. Moreover, recombinant fusion protein of HSP65 BCG plus a variable-number tandem repeats of MUC1, inoculated both as preventive and therapeutic treatment in mice, inhibits EL4 thymus lymphoma MUC1-expressing EL4 thymus lymphoma tumors and significantly prolonged the survival compared to non-vaccinated tumor-bearing mice [56]. BCG extracts have also been tested for lymphoma treatment. Lipomannan (LM) from BCG Tokyo was able to trigger antitumor effect in A20 tumor-bearing mice, contrary to CW extract or lipoarabinomannan antigen. Effect of LM from BCG seemed to be due to both recruitment of DCs that trigger superoxide production by eosinophils and memory Th2 cells [161]. In another study, maturation of DCs, specific cytolytic T cell activity, and antitumor effect in an ovalbumin (OVA)-expressing EL4 tumor model were seen combining Tokyo BCG strain-CWS formulated in nanoparticles with OVA-loaded nanoparticles [162].

## 9. Sarcoma and Mesothelioma and Mycobacteria

Inoperable sarcoma was the focus of Coley’s studies back in the 19th century, and more recently, several polysaccharides from BCG have shown to have antitumor effect on murine sarcoma models. In 1994, Lou et al. formulated PS1, a complex polysaccharide derived from BCG, into gelatin particles; however, no increased antitumor activity with respect to unencapsulated PS1 was observed, neither against S180 murine sarcoma cells in vitro nor in the mice model [166]. Boiling-water extracts mainly containing glucose and high-molecular-weight polysaccharide glycans from BCG Connaught [168], BCG Tice [169], and *M. vaccae* [167,170] demonstrated antitumor activity against murine sarcoma model. Furthermore, products from the digestion in urea of BCG Connaught [168] and *M. vaccae* [170] were also active against sarcomas. Surprisingly, the fractions obtained from *M. vaccae,* although active in the murine model, showed no direct antitumor effect in vitro. The authors further studied this phenomenon and designated the macrophages as responsible for the positive immune response orchestrated around the tumor [167].

Meth A fibrosarcoma-bearing mice treated either with radiation or Z-100 experienced a slight tumor inhibition, but when used in combination, the inhibition was improved [92]. Another successful case of Z-100 combined therapy is the administration together with *Corynebacterium parvum*, which not only made tumors completely regress but also prevented tumor growth if animals were re-challenged with more tumor cells [165].

As regards mycobacterial proteins, fusion proteins between PSA and *M. tuberculosis* HSP70 or *M. bovis* HSP65 activated cytotoxic T cell responses, which prevented the implantation of PSA-expressing-MC57 fibrosarcoma cells inoculated in mice. However, no significant differences were observed between the fused plasmid and the single protein PSA plasmid; thus in this case, the presence of the mycobacterial proteins did not contribute to an enhanced cytotoxic response or give an extra tumor-protective advantage [54].

As mentioned before, live BCG or BCG extracts have been widely used as adjuvant therapy in osteosarcoma animal models and patients, and it is known that BCG Connaught exerts its antitumor activity mediated by fibronectin attachment to sarcoma cells [171]. However, a case report of cutaneous TB in a patient with pelvic osteosarcoma treated with live BCG discourages its use due to the immunocompromising status of patients who undergo chemotherapy treatment [172]. Unlike using live BCG, promising results have been obtained in the use of L-MTP-PE. L-MTP-PE lowered the risk of recurrences and death in osteosarcoma patients [71] and eradicated lung metastasis [164]. Moreover, L-MTP-PE in combination with zoledronic acid (a potent inhibitor of bone resorption) showed promising results in xenogeneic and syngeneic mice models of osteosarcoma. The combination therapy induced an additive, and in some cases synergistic, inhibition of primary tumor progression [164,173]. Clinical I and II phases demonstrated the monocyte-mediated cytotoxic activity and an increased production of TNF-α and IL-6 cytokines. Later, a phase III study corroborated the previous results, showing increased overall survival of patients with predominantly non-metastatic and resectable tumors. Due to the satisfactory results and good tolerability of the drug, this treatment was approved and is currently being used in Europe (reviewed in [173]).

As regards mesothelioma, the same positive results seen in ovarian cancer (see Section 7) using HSP70 fused to an antibody fragment directed at mesothelin were also observed in malignant mesothelioma-bearing mice [148]. Two studies are based on the intradermal administration of SRL172 in combination with chemotherapy. In the first small clinical trial, a trend towards improved response rate was found in patients receiving both therapies [105]. In the second study, a phase I trial was carried out with intradermal and intratumor SRL172 injection together with chemotherapy. No toxicity was found, and the appropriate dose was established for a further phase II study [163]. It is worth noting that no more studies have been published on this issue.

## 10. Conclusions

When mycobacterial agents used for the treatment of non-urological cancers are revised, it can be observed that many laboratories use the whole mycobacterium, mainly in the heat-killed form. Thus, the structural molecules located in the mycobacteria cell wall could be crucial to trigger a favorable antitumor immune response, but also could be relevant for hampering the interaction of other key antigens with the immune system. The presence of some molecules in the cell wall can disturb the immunomostimulatory capacity of antigenic mycobacterial components. For instance, the smooth morphotype of *M. vaccae*, which possesses a polyester in its cell wall, induces an anti-inflammatory response to compare to the pro-inflammatory profile triggered by the rough morphotype which devoids the polyester [30]. Similarly, BCG substrains differ in their capacity to trigger cytokine production in cancer or immune cells [41,50,174]. The use of different BCG substrains for non-urological cancers is worthy of mention as detailed through this review since it could influence on the reported outcomes as antitumor agent due to their different antigenic profile. Thus, the knowledge of the repertoire of antigenic compounds and their location in each species is relevant for the design of specific therapies for each type of cancer.

Other mycobacteria molecules located inside the cell or excreted by the cell, such as Ag85 complex proteins [175] or heat-shock proteins have also been described as potent activators of the immune system. In fact, a great part of the works described in this review are based on the use of different mycobacterial HSPs as vaccine adjuvants. HSPs are able to activate antigen-presenting cells such as dendritic cells, to trigger the production of chemokines and cytokines after binding to different Toll-like receptors; and HSPs by themselves can act as adjuvants due to their capacity to deliver peptides that are recognized by the major histocompatibility complex, priming the adaptative immunity [176,177,178,179]. Previous studies have demonstrated the role of heat-killed BCG and *M. vaccae* to prime the immune system for diverse proteins [178,180]. Therefore, the potential to manipulate the tumor environment with mycobacterial HSPs when administrated together with tumor antigens or even with the whole heat-killed mycobacterium could be considered in the rational design of mycobacteria-based improved immunotherapies.

The efficacy of mycobacterium species as immunomodulatory agents is unquestionable. The abovementioned BCG and other mycobacteria species have demonstrated a role in the treatment of different urological cancers, but, outstandingly, no effect of some of these species was reported when evaluated in other types of cancers. This detailed knowledge of the mechanism by which mycobacteria are able to modify tumor microenvironment could even enable the physician to decide the ideal treatment according to the patient conditions. Our research efforts should be addressed to the knowledge of the key immunostimulatory mycobacterial antigens or the rational combination of certain mycobacterial antigens; the study of the host immune response, including also the host genetic background; the knowledge of the tumor microenvironment in each status of the disease; and the finding of appropriate biomarkers to be able to measure the efficacy of the mycobacterial immunotherapy effect in each patient. In view of all that explained above, the link between mycobacteria and cancer treatment has a lot of room for improvement.

## Figures and Tables

**Table 1:** Promising mycobacteria and mycobacteria components studied in vivo and in clinical trials to treat several types of cancer.

Type of Cancer	Species	Mycobacteria or Mycobacteria Components	In Vivo Experiments Outcome	Clinical Trials Outcome
**Breast Cancer**	*M. bovis* BCG	BCG ^/MUC1/ IL-2^	Inhibit tumor growth [133]	
		BCG ^/MUC1/GM-CSF^	Inhibit tumor growth [134]	
		BCG ^/MUC1/CD80^	Elicit tumor-specific immune response [135]	
		BCG + Allogenic tumor cells		Increase Survival [136]
		Tumor cells + BCG + Formalin	Safe and no toxic [137]	Safe, Increase Survival [137]
		HSP65	Immunogenicity, induce humoral response [139,140]	
		HSP65 + MUC1	Safe [141]	
	*M. phlei*	CW + Photodynamic	Trigger immune response [143]	
	*M. indicus pranii* (MIP)	MIP + Survivin + Alum	Immunogenicity and tumor growth inhibition [144]	
		MIP + 1’-S-1’-acetoxychavicol acetate + Cisplatin	Control cancer progression [145]	
	*M. smegmatis*	*M. smegmatis* + Monosodium urate crystals	Trigger immune response, delay the tumor [86]	
**Cervical Cancer**	*M. tuberculosis*	HSP70 + E7	Specific CD8+ T Cell Responses and Antitumor Effect [150]	
	*M. bovis* BCG	HSP65 + E7	Reduce carcinoma [151]; regression of palpable tumors, increase long-term survival [152]	Tumor regression [154,155]
	*M. indicus pranii* (MIP)	HK-MIP + HPV16T epitope	Cytotoxic T lymphocytes cytolysis [153]	
**Colorectal Cancer**	*M. tuberculosis*	Rv2299c (HSP90 family) + Tumor Antigen-activated DC	No generation of immune-suppressive cells, induce Th1 immune response [121]	
	*M. paragordonae*	HK- *M. paragordonae* + cisplatin	Induce antitumor immune response [122]	
**Esophageal cancer**	*M. smegmatis*	*M. smegmatis* ^MAGEA3/ SSX2^	Reduce tumor volume [111]	
**Fibrosarcoma**	*M. tuberculosis*	Z-100 + Radiation	Inhibit tumor growth [92]	
	*M. bovis* BCG	HSP65 + PSA	Protect to PSA-expressing tumors [54]	
**Gastric Cancer**	*M. bovis* BCG	BCG + 5-Fluorouracil, doxorubicin, and mitomycin		Improve survival [112]
**Hepatoma**	*M. tuberculosis*	HSP70 + Human umbilical vein endothelial cell	Inhibit tumor growth, prolong survival [124]	
	*M. bovis* BCG	GnRH(3)-hinge-MVP-Hsp65	Decrease tumor weight [125]	
		BCG + Proton-beam radiotherapy		Safe [132]
		HSP65-X10-beta-hCGCTP37	Inhibit tumor growth [126]	
		BCG 64-kDa surface protein	Inhibit tumor growth [127]	
		CWS of BCG (SMP-105)	Tumor-eliminating effect [129,130]	
		DNA + Poly-L-lisine	Delay of tumor growth [131]	
**Leukemia**	*M. tuberculosis*	HSP70	Prolong survival [157]	
	*M. bovis* BCG	CWS + WT1	Specific immune responses [95]	
**Lung Cancer**	*M. tuberculosis*	Z-100 + Radiation	Inhibit tumor growth [92]	
	*M. bovis* BCG	BCG + maltose-binding protein from *E. coli*	Induce Th1 response [99]	
		CWS	Reducce metastasis [75]	
		CWS + WT1	Reject of WT1-expressing lung cancer cells [95]	
		SMP-105 + mitomycin C-inactivated tumor cells	Supress tumor growth [94]	
	*M. vaccae*	SRL172		Improve quality of life [107]
		SRL172 + chemotherapy		Improve quality of life and survival [108,109]
	*M. indicus pranii* (MIP)	MIP + Cisplatin + Radiotherapy		Improve quality of life, regress tumor size [101]
**Lymphoma**	*M. tuberculosis*	HSP70 + Idiotype	Prolong survival [159]	
		HSP70 + A20 tumor cells	Regress tumor size [160]	
	*M. bovis* BCG	HSP65 + MUC1	Specific CTL and anti-tumor responses [56]	
		CWS in nanoparticles	Inhibit tumor growth, induce cytotoxic T cells [162]	
		Lipomannan	Inhibits tumor growth, eosinophils infiltration [161]	
**Melanoma**	*M. bovis*	Liposomes with CpG oligodeoxynucleotides	Increase natural killer and CD8(+) T cells, reduce regulatory CD4(+) T cell recruitment [58]	
	*M. tuberculosis*	B16F10/ESAT-6-GPI-IL-21	Inhibit tumor growth, prolong survival [62]	
		B16F10-ESAT-6-gpi/IL-21	Inhibit tumor growth [63]	
		ESAT-6 + IL-2	Tumor regression [64]	
		HSP70 + B16 cells lysate	Inhibit tumor growth, prolong survival [51]	
		HSP70 + Thymidine kinase genes (HSV) + attenuated *S. typhimurium*	Suppress tumor growth and extend survival [52]	
	*M. bovis* BCG	BCG + MUC1 + MBP	Inhibit tumor growth, induce Th1 response [74]	
		CpG oligodeoxynucleotide + HSP65 + MUC1	Inhibit tumor growth, prolong survival [57]	
		CWS	Inhibited tumor metastasis [75]	
	*M. vaccae*	SRL172		Increase survival [80]
	*M. obuense*	IMM-101	Reduce metastatic lesions [81], safe [82]	
	*M. indicus pranii* (MIP)	MIP	Reduce tumor growth and weight [77,84], block tumor growth and inhibit metastasis [85]	
	*M. smegmatis*	*M. smegmatis* + Monosodium urate crystals	Delay subcutaneous melanomas [86]	
**Mesothelioma**	*M. tuberculosis*	HSP70 + single-chain antibody	Increase survival, slow tumor growth, augment tumor-specific CD8+ T-cells [148]	
	*M. vaccae*	SRL 172 + Chemotherapy		Safe [163]
**Myeloma**	*M. indicus pranii* (MIP)	MIP	Reduce tumor growth [158]	
**Oral melanoma**	*M. bovis* BCG	L-MTP-PE	Prolong survival [72]	
**Osteosarcoma**	*M. bovis* BCG	L-MTP-PE		Decrease risk of recurrence and death [71]
		L-MTP-PE + Zoledronic acid		Inhibit primary osteosarcoma progression [164]
**Ovarian cancer**	*M. tuberculosis*	HSP70 + single-chain antibody	Increase survival, slow tumor growth, augment tumor-specific CD8+ T-cells [148]	
	*M. bovis* BCG	CWS		High quality of life [146]
**Pancreatic cancer**	*M. bovis* BCG	HSP65 + Tumor tissue lysate of pancreatic cancer	Prolong survival [114]	
	*M. obuense*	IMM-101 + Gemcitabine		Improve overall survival [115]
**Sarcoma**	*M. tuberculosis*	Z-100 + *C. parvum*	Prolong concomitant antitumor immunity [165]	
	*M. bovis* BCG	PS1 glycan	Suppress tumor cell growth [166]	
	*M. vaccae*	PS4A proteoglycan	Decreased tumor incidence [167]	
**Thymoma**	*M. indicus pranii* (MIP)	MIP	Reduce tumor growth [158]	

Ag85—antigen 85 complex, BCG—*Mycobacterium bovis* bacillus Calmette-Guerin, CpG—cytosine-guanosine nucleotide, CW—cell wall, CWS—cell wall skeleton, DC—dendritic cell, GPI—glycosyl-phosphatidylinositol, HK—heat-killed, HSP—heat-shock proteins, HSV—herpes simplex virus, HPV—human papillomavirus, IL-—Interleucin, IMM-101—heat-killed *Mycolicibacterium obuense*, L-MTP-PE—liposomal muramyl tripeptide phosphatidyl ethanolamine, MIP—*Mycobacterium indicus pranii*, MUC1—tumor-associated antigen Mucin 1, PSA—prostate-specific antigen, SRL 172—*M. vaccae* commercial preparation, Rv2299c—Mycobacterium tuberculosis HSP90 family member, SMP-105—CWS-BCG Tokyo 172, WT1—Wilms’ tumor protein, Z-100—an arabinomannan from *M. tuberculosis* strain Aoyama B.
